# Self-Reported Juvenile Firesetting: Results from Two National Survey Datasets

**DOI:** 10.3389/fpubh.2013.00060

**Published:** 2013-12-09

**Authors:** Carrie Howell Bowling, Joav Merrick, Hatim A. Omar

**Affiliations:** ^1^Lexington Fire Department, Fire Investigation Bureau, Lexington, KY, USA; ^2^Division of Adolescent Medicine, Department of Pediatrics, UK Healthcare, University of Kentucky College of Medicine, Lexington, KY, USA; ^3^National Institute of Child Health and Human Development, Jerusalem, Israel; ^4^Office of the Medical Director, Health Services, Division for Intellectual and Developmental Disabilities, Ministry of Social Affairs and Social Services, Jerusalem, Israel; ^5^Division of Pediatrics, Hadassah Medical Center, The Hebrew University of Jerusalem, Jerusalem, Israel

**Keywords:** firesetting, firesetter, academic performance, juvenile, attention deficit, behavior, adolescence, public health

## Abstract

The main purpose of this study was to address gaps in existing research by examining the relationship between academic performance and attention problems with juvenile firesetting. Two datasets from the Achenbach System for Empirically Based Assessment (ASEBA) were used. The Factor Analysis Dataset (*N* = 975) was utilized and results indicated that adolescents who report lower academic performance are more likely to set fires. Additionally, adolescents who report a poor attitude toward school are even more likely to set fires. Results also indicated that attention problems are predictive of self-reported firesetting. The National Survey Dataset (*N* = 1158) was analyzed to determine the prevalence of firesetting in a normative sample and also examine whether these children reported higher levels of internalizing and externalizing behavior problems. It was found that 4.5% of adolescents in the generalized sample reported firesetting. Firesetters reported more internalizing, externalizing, and total problems than their non-firesetting peers. In this normative sample, firesetters were found to have lower academic performance and more attention problems. Limitations include the low overall number of firesetters in each dataset (Factor Analysis *n* = 123 and National Survey *n* = 53) and the inclusion of children who had been referred for services in the Factor Analysis Dataset.

## Introduction

In 2001, the United States Fire Administration published findings indicating that an average of 3650 children aged 14 years and younger were injured or killed in fires each year. A previous study by the National Fire Protection Association reported that one-third of all children who died in fires had set the fire that killed them (1). Based on these statistics, it can then be estimated that over 1200 children each year are killing themselves through inappropriate use of fire. In comparison, the Children’s Defense Fund reported in 2009 that 938 children were killed by firearms accidentally or by suicide (2). Unfortunately firesetting does not receive the same media attention as gun violence and deaths. Juveniles are arrested for arson more than any other crime. The Office of Justice Programs reports that in 2006, 49% of the individuals arrested for arson were under the age of 18 years (3).

## Review of Literature

Age and gender are consistently found to be significant predictors of firesetting behavior with boys of all ages more likely to set fires than their female counterparts.

Male gender is highly associated with firesetting. Across studies, firesetting is more prevalent in boys than girls with rates as high as 69–91% in some samples (4–10). A study of 18-year-old males and females found that 70% of the males reported playing with fire in childhood and over half reported they played with fireworks. In comparison only 44% of the females reported firesetting or fire play (11). Boys are also more likely to set multiple fires (12).

A child’s age has been shown to be associated with the type of firesetting behavior exhibited and fire play has been found to correlate with developmental age ranges as well. Interest in fire typically is exhibited in children 3- to 5-years-of-age. Firesetting at this age may not be cause for panic as it can be part of a child’s normal curiosity (13). Clinical studies of juvenile firesetters confirm that many children had set their first fire, also known as their index fire, when they were between 6- and 8-years-old (14, 15) and the average age of many firesetters involved in fire education programs, residential treatment, or psychiatric hospitals is 9-years-old (5, 7, 16). According to Showers and Pickrell (12), the “youngest group of firesetters ages 4–8 was significantly more likely to set fires with financial cost of $500.00 or higher” (p. 496). Other studies also indicate a high percentage of children identified as firesetters are below 12-years-old with a smaller percentage of children falling into the older adolescent age range (12). Older age is associated with a child being more likely to seek out ignition materials and also re-offend [Ref. (7), p. 119]. Unfortunately, data are limited on children over 12-years-of-age who have set fires. Many jurisdictions set 12-years-of-age as the cut-off for charging children with arson. At this age, children can be remanded to court and enter the juvenile justice system versus the mental health or community services systems.

### Behavioral and psychological characteristics

Children displaying fire play and firesetting behavior exhibit a wide variety of behavioral and psychological problems. Even when comparing firesetter and non-firesetter groups within inpatient, mental health, and hospital settings differences have been identified. A relationship has been found between conduct problems, delinquency, and attention deficit/hyperactivity (ADHD) symptoms and firesetting.

Conduct disorder and other externalizing behaviors, such as aggression and delinquency; have been shown in numerous studies to correlate with firesetting (10, 16–23). Compared to gender-matched controls and control groups, a larger percentage, ranging from 60 to 64.5%, of juvenile firesetters were diagnosed with conduct disorder than their peers (12, 17). In one study (*N* = 204), 76.9% of children in a psychiatric outpatient center with a diagnosis of conduct disorder exhibited firesetting behavior (13). Several researchers (10, 14–16) studied children identified on a continuum of firesetting from severe to no firesetting at all. All of these studies found that a diagnosis of conduct disorder was correlated with higher levels of firesetting behavior.

Children who set fires are also more likely to exhibit problematic and antisocial behaviors such as aggression, delinquency, stealing, and truancy. Within inpatient and hospitalized samples, firesetters, and children who played with fire were distinguished by higher scores on aggression and hostility factors (6) and also received more reports of aggression, delinquency, and cruelty (19). When comparing children divided into groups by their firesetting status (non, severe, and minor) several studies found higher levels of poor social skills and social judgment to be related to firesetting behavior (10, 15, 19, 21).

A relationship between antisocial behavior and firesetting exists even when controlling for conduct problems (14). Studies conducted within community populations also support the finding that antisocial behavior is a strong predictor of firesetting. Martin et al. (8) reported the odds of a juvenile with serious antisocial behavior setting a fire was seven times greater compared to a child who exhibits a low number of antisocial behaviors. Children and adolescents who set fires are also more likely to be involved with illegal drugs and display risk-taking behavior.

Children who set fires exhibit more internalizing behaviors than their peers. Kolko and Kazdin (6) found that firesetters and match players received higher internalizing scores on the Achenbach child behavior checklist (CBCL) when compared with inpatient cases, and the firesetting group rated internalizing problems higher than the other two groups. Self-injurious behavior, suicidal thoughts, and suicide attempts are also found in higher rates among juveniles reporting involvement with fire and matches. In the same study both firesetters and match players received higher scores on self-injury measures than children who never played with fire or matches. Martin et al. (8) found that firesetters report more suicidal thoughts when compared to peers who report no firesetting or fire play. In a study comparing juvenile arsonists and juvenile criminals, 74% of the arsonists reported suicidal thoughts, and 44% reported attempting suicide (9).

### School functioning and attention characteristics

Cognitive, academic, and attentional characteristics also differentiate children and adolescents who set fires from their non-firesetting peers. Unfortunately, information on the cognitive functioning and academic performance of juvenile firesetters is more limited than research into the behavioral and psychological functioning of these children. Intelligence as measured by general intelligence tests does not appear to differentiate firesetters from non-firesetters, in samples from clinics, school populations, and random samples from the community (7, 18, 24, 25). Components of cognitive functioning, such as poor planning ability and poor understanding of cause and effect relationships, however, are associated with children who play with fire (15). In our experience, many children who set fires report that they did not expect the fire to spread or grow so quickly. Additionally, both younger children and adolescents say they did not think through what they would do to put the fire out prior to setting it. Their responses are representative of these facets of cognitive functioning.

Firesetters differ from other groups of children on school and academic performance in the few studies conducted utilizing school information. Firesetters and delinquent control groups are shown to have “poor academic performance, history of grade failure, and truancy” [Ref. (12), p. 498]. Firesetting is a specific type of delinquent behavior; and, therefore it is not surprising that children who set fires have similar struggles in school as their delinquent peers.

Children who set fires also have a higher incidence of ADHD. Studies suggest that the associated impulsivity plays a role in a juvenile’s ability to inhibit their behavior and contributes to playing with lighters, matches, and firesetting. When comparing firesetters and non-firesetters, juvenile firesetters with impulsive behavior had less inhibition when compared to non-firesetters in a residential placement (15). Additionally, firesetters and children who played with matches have been rated higher in “emotionality, impulsivity, and lower sociability than non-firesetters” [Ref. (6), p. 196]. Impulsivity also differentiates between firesetting groups based on severity with more severe firesetters and more persistent firesetters exhibiting more impulsivity (10). Of the juveniles referred to a fire setter intervention program in San Diego County, California “between 20 and 40% of the children had been diagnosed with attention deficit disorder (ADD) or exceed[ed] the criterion in the Diagnostic Statistical Manual – Fourth Edition” (26), while one study (12) found only 20% of firesetters received a diagnosis of ADD. Further research into the correlation of firesetting and ADD/ADHD would be beneficial to determine the extent that impulsivity plays a role in children’s firesetting behaviors. It would also be helpful to determine if management of ADD/ADHD symptoms would minimize firesetting as well.

### Gaps in the research

Many specific facets of cognitive functioning have been cited as contributing to firesetting behavior, however, little research has specifically looked at overall cognitive abilities of these children, and even fewer studies have investigated academic and school functioning. A handful of researchers (7, 18, 24, 25) have investigated differences in the overall intelligence quotients between firesetters and non-firesetters in samples from clinics, school populations, and community venues. These studies found no difference in overall cognitive functioning. Other studies pulling out aspects of cognitive ability such as formal operations, planning ability, and understanding of cause and effect relationships do reveal differences between children identified as firesetters and those who had not set fires (15, 27). The scarcity of such studies is a significant gap in the research on firesetting.

Even less is known about how children who set fires perform academically. Showers and Pickrell (12) found that both firesetters and children in a delinquent control group exhibited poor academic performance, a history of failing grades, and truancy. Two studies by Kafry (18) and Kolko and Kazdin (6) found that firesetters can be differentiated from their peers academically and have depressed social skills and behavior problems. These limited studies suggest that firesetters are differentiated from other children in the classroom, just as other children with behavioral challenges can be identified. Unfortunately, with the exception of these few studies, little has been done to assist teachers in identifying a child who is at-risk for firesetting in the same way efforts have been taken to identify children at-risk for other types of violence. This is very unfortunate given that during the 2003–2005 school year, 14,700 fires that required the fire department to respond occurred on school properties (28).

Studies conducted with large samples populations are rare. Of the studies examined, the majority utilized sample sizes of <200 individuals, ranging from 17 to 192 (14, 29). Only three had datasets contained more than 1000 individuals (8, 25, 30).

## Purpose of this Study

The purpose of this study was to examine the characteristics of children who set fires and then further identify if school related variables are predictive of this behavior. The academic and school functioning of children identified as firesetters has only been minimally researched and therefore discovering differences in the academic and school functioning of self-reported firesetters and non-firesetters would be relevant for teachers and school-based mental health practitioners. Although ADHD and firesetting has been better studied, the findings are mixed. Further investigation of self-reported attention problems will lead to further understanding of whether impulsivity plays a role in firesetting. On a broader scope, the true prevalence of juvenile firesetting behavior needs additional inquiry. Most fires set by children and adolescents are never reported to a fire department due to the parents not discovering the child’s behavior or caregivers choosing not to report this behavior to authorities.

## Materials and Methods

This study utilized existing data samples for the Achenbach System of Empirically Based Assessment (www.aseba.org). The ASEBA is used in a variety of settings, including schools, medical facilities, public health agencies, and other social and mental health services (www.aseba.org). Additionally, the ASEBA has been used in multiple studies on juvenile firesetting (25, 31–36). Several prominent manuals on juvenile firesetting also recommend the inclusion of the ASEBA report forms in the assessment of children who set fires (15, 37).

Firesetting behavior is addressed on the ASEBA Youth Self-Report (YSR) form completed by the juvenile and the CBCL form that is completed by the parent or guardian. This question appears as item #72 “I set fires” and “sets fires” on the two forms respectively. The directions indicate that the juvenile and parent/caregiver should rate firesetting behavior in the past 6 months so only recent firesetting behavior is captured. Item #72 is also considered a critical item that indicates a high risk or safety issue.

Several scales were used in this study including the Attention Problems, ADHD, Externalizing, Internalizing, and Total Problems scales. The raw scores for these scales were utilized in the analyses. The Manual for the ASEBA School-Age Forms and Profiles (38) recommends using the raw scores for research due to the way *T*-scores were assigned. There is a truncation of scores that are at or below the 50th percentile when the *T*-scores were developed (p. 89). This truncation results in a loss of differences among low scores since raw scores of 0, 1, 2, 3, and 4 may have a *T*-score of 50 on one scale and scores of 0 and 1 may have a *T*-score of 50 on another scale. Additionally, *T*-scores above 70, or 98th percentile were assigned with as many increments as possible given the raw scores obtained for each scale.

### Data sets

Two different data sets available from ASEBA were utilized for this study: the National Survey Data and the Factor Analysis Data. The National Survey dataset is data derived from the 1999 National Survey of Children, Youth, and Adults conducted by Temple University’s Institute for Survey Research. This data set was utilized to address secondary research questions 3 and 4 and investigate the prevalence of self-reported firesetting as well as some of the characteristics associated with this behavior in a larger sample.

The data set utilized to address the main research questions examining academics and attention is the 1999 Factor Analysis Data set and is derived from the National Survey population. The Factor Analysis set “consists of referred people and non-referred people with high Total Problem scores from the National Survey” [Ref. (38), p. 74]. In order to identify high scorers the median Total Problems score was identified for boys and girls in the 1999 National Survey sample. The children selected to be included in the Factor Analysis sample were those with total problems score above this median [Ref. (38), p. 82]. These “referred and non-referred people” consist of individuals pulled from the larger National Survey Data set and an additional group of youth from 13 outpatient and inpatient mental health services. Individuals included were from 40 US States, the District of Columbia, one Australian state, and England. The children from the National Survey which are included in the Factor Analysis Data Set received high Total Problem scores but may or may not be receiving services.

### Participants

The Factor Analysis dataset yielded 975 matched cases (*N* = 975) with responses from the youth and the caregiver/guardian (see Table 1). The National Survey dataset consisted of 1158 matched cases (*N* = 1158) (see Table 2).

**Table 1:** **Descriptives for factor analysis sample**.

	Total	Firesetters	Non-firesetters	Males	Females	White	African American	Other race
*N*	975	123	852	579	396	381	164	301

**Table 2:** **Descriptives for national survey sample**.

	Total	Firesetters	Non-firesetters	Males	Females	White	African American	Other race
*N*	1158	53	1105	610	551	718	227	216

## Results

The primary questions of interest in this study relate to academic performance and attention. Specifically, are academic and attention problems predictive of firesetting?

### Variables for hypotheses 1 and 2 (factor analysis dataset)

The Achenbach CBCL parent and YSR are a rich source of data and specific variables were selected to look at academic performance and attention.

### Firesetting

Item #72 (I set fires) served as the dependent variable. The original range of possible responses to the question “I set fires” was a 3-point Likert scale ranging from 0 (not true) to 2 (very true or often true). Firesetting was recoded for this study to a dichotomous variable with 0 (no firesetting) and 1 (firesetting). This recode was done for several reasons. The original scale of this question hints at the severity of firesetting but does not give parameters; therefore, a score of 2 for one juvenile may not be as severe as a 2 rating for another juvenile. More importantly, it is this author’s opinion that any incident of firesetting can have severe consequences so the distinction between “somewhat or sometimes true” and “very true or often true” is irrelevant since any instance of firesetting or fireplay is dangerous. Children were coded as firesetters if they reported “somewhat” or “often” true that “I set fires.” Children were only coded as non-firesetters if they responded “0,” that they do not set fires. As expected, the majority of children and parents reported no firesetting behavior (*n* = 852 and *n* = 887, respectively) in the Factor Analysis sample. The adolescents self-reported more firesetting than their guardian/caregiver. Of the 123 children who did report setting fires, only 32 reported that the “I set fires” statement was “very true” of them. After recoding, over 12% of the total respondents reported some level of firesetting behavior (*n* = 123).

### Independent variables

The predictor variables for the first two research questions included demographic variables and the predictor variables of interest. Created scales representing academic performance and attitude toward school and variables measuring attention and ADHD symptoms were identified for inclusion in this study.

### Demographics

Demographic information utilized included age, gender, and race variables. In the factor analysis sample, 40.6% of the individuals were female and 59.4% were male (*n* = 396 and *n* = 579, respectively). Gender was recoded as 0 (female) and 1 (male). The original race variable consisted of six groups. This variable was recoded as (race) with three groups: Caucasian, African American, and other (*n* = 381, *n* = 164, and *n* = 301). See Table 3 for firesetting category by gender and race.

**Table 3:** **Frequency of firesetting by gender and race**.

Firesetting reported	Males	Females	Caucasian	African American	Other race
Yes (1)	99	24	40	13	45
No (0)	480	372	341	151	256

*Factor analysis sample*.

The YSR is utilized with children and adolescents age 11–18. In the Factor Analysis sample, the mean age was 13.63 (SD = 2.06). The mean age for females was 14.09 years (SD = 2.16) and the mean age for boys was 13.32 years (SD = 1.93).

The raw data received from ASEBA did not contain any scales scores for academic performance or overall competence. Several scale reliability analyses were conducted to identify items that represented academic performance with good reliability prior to creating the final scale for academic performance. Eight items were included in the final academic performance scale, four from the YSR and four from the CBCL. These items rated a child’s academic performance in Language Arts/Reading/English, History/Social Studies, Arithmetic/Math, and Science. The rating scale is a Likert scale ranging from 1 (failing) to 4 (above average). Prior to creating the scale, each item was reverse coded with the range being 1 (above average) to 4 (failing). This recode was completed so the direction of the scale was consistent with other scales in the ASEBA data where a higher value represents more problems or negative symptoms. The Cronbach’s α for the created Academic Performance scale is 0.87. Both the child and parent ratings were included since the Cronbach’s α for the scale decreased if any item was deleted. Reference Table 4 for descriptives of the Academic Performance scale. This scale rates a child’s academic performance as measured by their grades in the main subject areas.

**Table 4:** **Descriptives for predictor variables in academic regression**.

	Age in years	Academic performance	Attitude toward school
*N* valid	975	704	975
*N* missing	0	271	0
*M*	13.63	2.16	0.60
SD	2.06	0.63	0.44

*Factor analysis sample*.

### Attitude toward school

This scale was created after examining item groupings for the Academic Performance Scale. Six items specifically grouped together to measure a child’s general demeanor or attitude toward school such as “My school work is poor,” “I cut classes or skip school,” and “I disobey at school.” These items are again rated on the same Likert scale from 0 (not true) to 2 (very true or often true), with higher values representing a more negative construct. Although these items do not measure a child’s grades, they assess another facet of a child’s performance at school and therefore this scale was included for additional analysis. The Attitude toward School scale (School Attitude) has a Cronbach’s α of 0.70. See Table 4 for additional descriptive information for this scale.

### Attention problems

The Attention Problem scale (Attention) consists of items such as “fails to finish,” “can’t sit still,” and “poor school work.” The raw score for this scale was utilized for hypothesis two to look at attentional problems that related to school functioning and may also contribute to firesetting. Both the YSR Attention Problems (*N* = 974, *M* = 8.03, SD = 3.09) and CBCL Attention Problems (*N* = 974, *M* = 8.55, SD = 4.43) raw scale scores were used. The CBCL has a Pearson (*r*) of 0.92 and Cronbach’s α of 0.86. The YSR has a Pearson (*r*) of 0.87 and Cronbach’s α of 0.79 [Ref. (38), p. 101]. Reference Table 5 for a summary of the descriptives for these scales.

**Table 5:** **Descriptives for predictor variables of ADHD and attention**.

	Attention youth	ADHD youth	Attention parent	ADHD parent
*N* valid	974	974	974	974
*N* missing	1	1	1	1
*M*	8.03	6.85	63.99	6.74
SD	3.09	7.22	9.81	3.49

*Factor analysis sample*.

### Attention deficit/hyperactivity problems

This scale (ADHD) consists of items that are consistent with a DSM diagnosis of ADD or Attention Deficit Hyperactivity Disorder. High scores on this scale are suggestive of either ADD or ADHD. The raw scale scores from the YSR (*N* = 974, *M* = 6.85, SD = 2.69) and CBCL (*N* = 974, *M* = 6.74, SD = 3.48) were used for hypothesis two. The CBCL has a Pearson (*r*) of 0.93 and Cronbach’s α of 0.84. The YSR has a Pearson (*r*) of 0.86 and Cronbach’s α of 0.77 [Ref. (38), p. 101]. Please see Table 5 for a summary of the descriptives for these variables.

### Procedures to investigate academic performance and firesetting

The first hypothesis investigated if any academic performance (Academic Performance) differences exist between firesetters and non-firesetters and if a child’s academic performance is predictive of firesetting.

Preliminary analyses were run on the predictor variables to determine the relationship between the variables and the presence of any confounding variables. The variables to include in the model had already been identified based on the research and focus of this study so the purpose of these initial analyses were to gain a better understanding of the data prior to using the variables in the full regression model.

The main analysis was performed utilizing logistic regression to determine if academic performance is predictive of firesetting when controlling for demographic variables and with consideration of confounding factors.

A secondary analysis was conducted using the Attitude toward School scale in a logistic regression as the explanatory variable and then in a logistic regression controlling for attitude toward school. These additional logistic regressions were done to examine if a child’s truancy, disobedience, and perception of their academic grades was predictive of firesetting and then if academic performance was predictive of firesetting when controlling for demographic variables and the child’s attitude toward school.

### Results of analyses examining academic performance and firesetting

The first research hypothesis addressed the relationship between academic performance and firesetting. It was expected that academic problems would be predictive of firesetting but to what extent poor school performance would increase the likelihood of firesetting was unknown. Also unknown was whether poor attitude to school would predict firesetting.

Preliminary analyses were conducted to determine if all initially selected variables should be included in the regression. The first variable to be examined was gender (Gender). When comparing males and females utilizing independent samples *t*-tests to compare means on the academic performance (Academic Performance) variable, it was found that boys have poorer academic performance (*M* = 2.24, SD = 0.62). The difference was statistically significant at the *p* < 0.001 level. A logistic regression was then run to examine the relationship between gender and firesetting. The odds of firesetting decrease by 69% if a child is female, compared to a male. This was significant at the *p* < 0.001 level. This information indicated that gender is associated with both academic performance and firesetting and therefore it was determined that it was appropriate to include gender in the regression as planned.

The race variable (Race) was also analyzed separately in relation to academic performance (Academic Performance) and firesetting. A one-way ANOVA revealed significant differences between the groups, *F*(2, 682) = 14.47, *p* < 0.001. Caucasian children reported higher academic performance and children who were not Caucasian or African American reported the worst academic performance. A logistic regression for race (Race) and firesetting (Fires) revealed no statistically significant difference (*p* = 0.08) between the three groups. Due to the relationship between race and academic performance, race was included in the regression model as a control variable.

**Figure 1:**
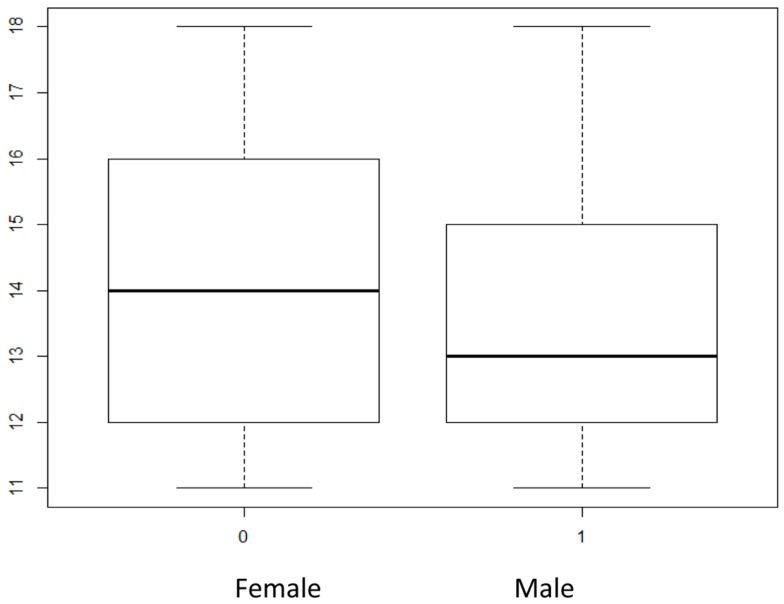
**Box plots of gender (x-axis) and age (y-axis) in factor analysis dataset**. Age distribution of males and females within the factor analysis sample. There were more older females than males in the sample.

A youth’s age was also examined in relationship to academic performance and firesetting. A one-way ANOVA revealed no statistically significant difference between age groups when looking at academic performance reports (Academic Performance) *F*(7, 696) = 1.17, *p* > 0.05. A logistic regression for the age and firesetting was then conducted. The odds of a child reporting firesetting behavior are 0.92 times less for every 1 year increase in the child’s age; however, this was not statistically significant (*p* = 0.08). Although this result was not significant at the *p* < 0.05 level, the low significance level (*p* = 0.08) was unexpected given what is known about age as it relates to firesetting behavior. Further analysis was done examining the age variable. Since this sample contains adolescents age 11–18 years-old the relationship between gender and age was examined to determine if the males and females were equally represented across ages. An independent samples *t*-test revealed that the females were a little older on average (*M* = 14.09, SD = 2.162) than males (*M* = 13.32, SD = 1.96) in this sample (see Figure 1). This difference was significant *t*(973) = 5.84, *p* < 0.001. The determination was made to exclude age from the predictors included in the full logistic regression because the association between firesetting and age in this sample was a function of gender, which is known to be predictive of firesetting.

The relationship between an adolescent’s academic performance and attitude toward school was also examined to further determine if this scale should be included in the regression model. As expected a child’s attitude toward school (School Attitude) and academic performance (Academic Performance) were significantly correlated with *r* = 0.583, *p* < 0.01. Although there is a strong correlation between these two variables, the determination was made to include the Attitude toward School variable in the regressions because this scale measures a different aspect of academic performance. The main focus of this study was to address gaps in the research and it is this author’s opinion that the Attitude toward School scale captures another important part of a child’s functioning at school.

The main analyses included three separate regressions to fully explain the relationship between academic performance and firesetting. The first regression was run with race and gender as controls to determine if academic performance (as measured by grades) was predictive of firesetting. Attitude toward school was included in a second regression as the explanatory variable and a third regression as a control to determine if academic performance was predictive of firesetting even when controlling for demographics and attitude.

$$\begin{array}{llll} & \mathrm{Race}\;+\;\mathrm{Gender}\;+\;\mathrm{AcademicPerformance}\to \mathrm{Firesetting}\; & & \;\\ & \mathrm{Race}\;+\;\mathrm{Gender}\;+\;\mathrm{SchoolAttitude}\to \mathrm{Firesetting}\; & & \;\\ & \mathrm{Race}\;+\;\mathrm{Gender}\;+\;\mathrm{SchoolAttitude}\; & & \;\\ & \;+\;\mathrm{AcademicPerformance}\to \mathrm{Firesetting}\; & & \; \end{array}$$

Results of the initial logistic regressions indicate that academic performance was a significant predictor of firesetting behavior (*p* < 0.05) when controlling for gender and race. When considering two children of the same race and gender, the odds of setting fires increases by 46% for every one unit increase in rating of poor academic performance on the Academic Performance scale (or a factor of 1.46), as can be observed in Table 6.

**Table 6:** **Logistic regression for academic performance. and firesetting**.

Predictors	β	SE	Sig.	Exp (β)
Gender	−1.19	0.30	0.000***	0.30
White	–	–	–	–
African American	−0.64	0.40	0.107	0.53
Other	0.26	0.27	0.342	1.29
Academic performance	0.38	0.19	0.049*	1.46

*Controls are gender and race*.

*Factor analysis sample (*N* = 685)*.

*Block 1: Nagelkerke *R*^2^ = 0.08; χ^2^(3) = 26.63; *p* < 0.001*.

*Block 2: Nagelkerke *R*^2^ = 0.09; χ^2^(4) = 30.41; *p* < 0.001*.

*p ≤ 0.05; **p ≤ 0.01; ***p ≤ 0.001.

A regression was then run to examine whether attitude toward school alone was a significant predictor of firesetting. It was found that for every one unit increase in a child’s poor attitude toward school, the odds of being a firesetter increase by a factor of 3.4. Due to these results and the identification of school attitude as a predictor and also as a possible confounding variable, a logistic regression was run to determine if academic performance would remain a significant predictor even when controlling for attitude toward school.

School Attitude was entered as a control variable after race and gender to investigate whether controlling for an adolescent’s rating of truancy, disobedience and their view of their academic performance would effect the predictive ability of academic performance (as measured by a child’s grades.) The logistic regression results change when entering school attitude as a control variable and including academic performance as the explanatory variable. The significance of academic performance as a predictor of firesetting changes from *p* = 0.049 to *p* = 0.478 with an odds ratio change from 1.46 to 0.85 (see Table 7). When comparing two children of the same gender, race, and with the same reported attitude toward school, academic performance is no longer a significant predictor of firesetting.

**Table 7:** **Logistic regression for academic performance and firesetting controlling for attitude toward school**.

Predictors	β	SE	Sig.	Exp (β)
Gender	−1.00	0.31	0.001***	0.37
White	–	–	–	–
African American	−0.67	0.40	0.097	0.51
Other	0.20	0.28	0.469	1.22
School attitude	1.47	0.33	0.000***	4.34
Academic performance	−0.17	0.23	0.478	0.85

*Controls are gender, race, and school attitude*.

*Factor analysis sample (*N* = 685)*.

*Block 1: Nagelkerke *R*^2^ = 0.08; χ^2^(3) = 26.63; *p* < 0.001*.

*Block 2: Nagelkerke *R*^2^ = 0.14; χ^2^(4) = 49.48; *p* < 0.001*.

*Block 3: Nagelkerke *R*^2^ = 0.14; χ^2^(5) = 49.48; *p* < 0.001*.

*p ≤ 0.05; **p ≤ 0.01; ***p ≤ 0.001.

### Procedures to investigate attention problems, ADHD, and firesetting

The second research hypothesis addressed the relationship between a child’s firesetting behavior and attention problems. The ASEBA rating forms yield one scale for Attention Problems and another that measures clinical symptoms of ADHD. These scales are present on the YSR and Parent Rating forms. The ASEBA research consultant and the ASEBA Manual for School – Age Forms and Profiles (37) recommend utilizing the raw scores for research due to the way that raw scores were converted to *T*-scores. Correlations and descriptives were run to determine if the youth and parent scores were similar or varied significantly. The correlations were significant at *p* < 0.01, when comparing the individual scales across raters (ranging from *r* = 0.30 to 0.36).

Several initial analyses were run to look at the relationship between the control and predictor variables and firesetting in order to check for confounding variables and better understand the variables. Initial *t*-tests were run to examine gender differences in attention problems (Attention Probs_raw) and (ADHD_raw) using both the adolescent self-report and the parent rating.

Next, one-way ANOVAs were run to look at the relationship between race, attention problems, and ADHD symptoms. The relationship between race and firesetting had previously been examined during the initial analyses for hypothesis one so this process was not repeated.

Final logistic regressions were run to fully address hypothesis two. First, the child’s and parent’s ratings of attention problems were entered into a logistic regression model after controlling for demographics. The child and parent’s ratings of ADHD symptoms were also utilized in logistic regressions to determine if a child’s or parent’s ratings were more predictive of firesetting.

### Results of analyses examining attention problems, ADHD, and firesetting

The second research hypothesis addressed whether an adolescents attention problems and/or ADHD were predictive of firesetting. The results of the initial analyses to examine the relationships between the predictor variables and firesetting identified several confounding variables. The gender groups were compared on the four attention scales. Boys and parents of boys reported significantly more attention problems and ADHD symptoms than females (see Table 8). The determination was made to include gender as a control variable in these regressions due to the relationship between gender, attention problems, ADHD, and firesetting.

**Table 8:** **Comparison of attention problems and ADHD by gender**.

Scale	Sex	*N*	Mean	SD	Sig.
Attention problems youth	Female	395	7.73	3.00	0.012*
	Male	579	8.23	3.13	
ADHD youth	Female	395	6.74	2.59	0.302
	Male	579	6.93	2.75	
Attention problems Parent	Female	395	7.23	4.50	0.000***
	Male	579	9.45	4.16	
ADHD parent	Female	395	5.77	3.46	0.000***
	Male	579	7.41	3.35	

*Factor analysis sample*.

*p ≤ 0.05; **p ≤ 0.01; ***p ≤ 0.001

Race was also examined in relation to attention problems and symptoms of ADHD. There was a statistically significant difference in the rating of attention problems by the youth and the parent and also the parent’s report of ADHD symptoms. Caucasian adolescents reported more attention problems then their African American peers, *F*(2, 842) = 3.58, *p* < 0.05. Conversely, parents of African American adolescents reported more attention problems than parents of Caucasian children, *F*(2, 842) = 6.24, *p* < 0.01. They also reported more ADHD symptoms than parents of Caucasian and Other race children, *F*(2, 842) = 8.09, *p* < 0.001. Due to this finding, race was also included as a control variable.

Two logistic regressions were run using the ratings on the YSR and Parent rating of Attention Problems to predict the likelihood that a child with more reported attention problems would set fires. Both the child and parent report of attention problems (Attention raw and Attention Raw_CBCL) indicate that there is a significant relationship between attention problems and firesetting (*p* < 0.05). Based on the youth’s report, the odds are 8.0% higher of being a firesetter for every one unit increase in the attention problem raw score (see Table 9). Based on parental/caregiver reports, a youth’s odds of being a firesetter increase by 7.0% for every one unit increase in the attention problem raw score (see Table 10).

**Table 9:** **Logistic regression for self-reported attention problems and firesetting**.

Predictors	β	SE	Sig.	Exp (β)
Gender	−1.31	0.27	0.000	0.27***
White	–	–	–	–
African American	−0.39	0.34	0.258	0.68
Other	0.42	0.23	0.075	1.53
Attention problems	0.08	0.04	0.034	1.08*

*Controls are gender and race*.

Factor analysis sample.Block 1: Nagelkerke *R*^2^ = 0.08; χ^2^(3) = 36.23; *p* < 0.001.Block 2: Nagelkerke *R*^2^ = 0.09; χ^2^(4) = 40.73; *p* < 0.001.*p ≤ 0.05; **p ≤ 0.01; ***p ≤ 0.001.

**Table 10:** **Logistic regression for parent-reported attention problems and firesetting**.

Predictors	β	SE	Sig.	Exp (β)
Gender	−1.23	0.28	0.000	0.29***
White	–	–	–	–
African American	−0.51	0.34	0.136	0.60
Other	0.37	0.24	0.123	1.44
Attention problems	0.07	0.03	0.011	1.07*

*Controls are gender and race*.

*Factor analysis sample*.

*Block 1: Nagelkerke *R*^2^ = 0.08; χ^2^(3) = 36.23, *p* < 0.001*.

*Block 2: Nagelkerke *R*^2^ = 0.10; χ^2^(4) = 42.74; *p* < 0.001*.

*p ≤ 0.05; **p ≤ 0.01; ***p ≤ 0.001.

Two additional logistic regressions were run to investigate whether higher levels of reported ADHD symptoms would predict firesetting. Using the child and parent raw score on the ADHD variable (ADHD_rawscale and CBCLADHD_rawscale), the results indicated that the child’s rating of ADHD symptoms is not significantly predictive of firesetting (see Table 11). The parent/caregiver score was, however, significant at the *p* < 0.05 level. The odds of a child setting fires increases by 8.0% for every one unit increase in the ADHD raw scale score as reported by the parent (see Table 12).

**Table 11:** **Logistic regression for self-reported ADHD symptoms and firesetting**.

Predictors	β	SE	Sig.	Exp (β)
Gender	−1.34	0.27	0.000	0.26***
White	–	–	–	–
African American	−0.41	0.34	0.228	0.66
Other	0.41	0.29	0.087	1.50
ADHD	0.07	0.04	0.104	1.07

*Controls are gender and race*.

*Factor analysis sample*.

*Block 1: Nagelkerke *R*^2^ = 0.08; χ^2^(3) = 36.23; *p* < 0.001*.

*Block 2: Nagelkerke *R*^2^ = 0.09; χ^2^(4) = 38.85; *p* < 0.001*.

*p ≤ 0.05; **p ≤ 0.01; ***p ≤ 0.001.

**Table 12:** **Logistic regression for parent-reported ADHD symptoms and firesetting**.

Predictors	β	SE	Sig.	Exp (β)
Gender	−1.25	0.28	0.000	0.29***
White	–	–	–	–
African American	−0.51	0.34	0.133	0.60
Other	0.38	0.24	0.108	1.47
ADHD	0.08	0.03	0.016	1.08*

*Controls are gender and race*.

*Factor analysis sample*.

*Block 1: Nagelkerke *R*^2^ = 0.08; χ^2^(3) = 36.23; *p* < 0.001*.

*Block 2: Nagelkerke *R*^2^ = 0.10; χ^2^(4) = 41.99; *p* < 0.001*.

*p ≤ 0.05; **p ≤ 0.01; ***p ≤ 0.001.

### Results for the secondary research hypotheses

The National Survey sample dataset was utilized to examine the prevalence and characteristics of juvenile firesetting in a normative sample. As previously explained, the individuals in this dataset are much more diverse than many of the samples used in other research studies on firesetting.

### Variables for hypotheses 3 and 4 (national survey dataset)

Research questions 3 and 4 investigate the prevalence and characteristics of firesetters in the National Survey dataset, which is a more normative sample. Based on the purpose of these questions, variables were selected which have been investigated in other studies in order to allow comparison.

### Firesetting

Firesetting was also utilized as the dependent variable for hypotheses 3 and 4. As expected, the majority of children and parents reported no firesetting in the National Survey, which is a much more normative sample (see Table 13). Based on the same rationale discussed previously, the firesetting variable in the YSR data was recoded as 0 (no firesetting) and 1 (firesetting) (*N* = 1158). Only 53 adolescents reported any firesetting behavior, which is 4.6% of the total sample.

**Table 13:** **Frequency of adolescents and parents reporting firesetting in national survey sample**.

Response	Parent	Adolescent
Not true	1146	1105
Somewhat or sometimes true	14	47
Very true or often true	1	6
Missing		3

### Independent variables

The predictor variables for the secondary research questions also included demographic variables, several scale scores, and a created scale representing academic performance.

#### Demographics

Demographic information utilized includes age, gender, and race variables. In the National Survey dataset, 47.6% of the cases were female and 52.5% were male (*n* = 551 and *n* = 610, respectively). Gender was recoded with 0 (female) and 1 (male). The race variable was again recoded as (Race) with three groups; Caucasian, African American, and other (*n* = 718, *n* = 227, and *n* = 216, respectively). The age range of children and adolescents in the National Survey was also 11–18 years (*N* = 1161, *M* = 14.11, SD = 2.23). The mean age of the girls was 14.12 years and the mean age of the boys was 14.09 (see Table 14).

**Table 14:** **Descriptives for predictor variables in national survey sample**.

	Age	Internalizing problems	Externalizing problems	Total problems	Academic performance
*N* valid	1161	1159	1159	1159	1053
*N* missing	1	2	2	2	108
*M*	14.11	10.48	10.38	37.64	3.26
SD	2.23	62.46	7.49	22.32	0.49

#### Internalizing problems

The Internalizing Problems raw score (Internal_raw) from the YSR and CBCL were examined for use in hypothesis 4. The Internalizing grouping “mainly reflects problems within the self such as anxiety; depression; somatic complaints without known medical cause; and withdrawal from social contacts”. It was found that adolescents self-reported higher levels of internalizing problems (*N* = 1159). The self-reported mean of Internalizing Problems was 10.48 while parents reported a mean of 6.74. The determination was made to utilize the self-report score as it makes sense that the adolescents themselves are the best judge of their own thoughts and feelings. Reference Table 14 for descriptives of this scale.

#### Externalizing problems

The Externalizing Problems raw score (External_raw) was used for hypothesis 4 as well. The Externalizing Problem scale questions represent “conflicts with other people” and expectations for children’s behavior [Ref. (37), p. 93]. The means of the child and parent reported were examined.

Again, the mean for the self-report score was higher (*M* = 10.38) than the parent’s report of externalizing behavior (*M* = 7.77, *N* = 1160 for both groups). Consideration was given to the nature of the items in this scale and it also appears that the self-report score may provide a better gage of the child’s behavior. Many of the items refer to behavior that an older child or adolescent would hide from a parent including lying, sexual problems, Fighting, drug use, and drinking. See Table 14 for descriptives of the adolescent’s rating of Externalizing Problems.

#### Total problems

The Total Problems *T*-score (TotalProblems_raw) represents the child’s score on all the problem items. This scale score was used for hypothesis 4. The youth self-reported score was utilized. The mean and standard deviation is described in Table 14.

#### Academic performance

The same academic performance scale was created using the National Survey data. The Cronbach’s α for the scale (Academic Performance) was 0.84 (*N* = 1053). Reference Table 14 for descriptive information on this created scale.

#### Attention problems and ADHD

The Attention Problem scale (Attention) and ADHD scale (ADHD) raw scores were utilized in hypothesis 4. Reference Table 15 for descriptives of these four scales.

**Table 15:** **Descriptives of attention problems and ADHD symptoms**.

	Attention youth	Attention parent	ADHD youth	ADHD parent
*N*	1159	1160	1159	1160
*M*	5.12	3.89	4.59	3.19
SD	3.30	3.75	2.87	3.00

*National survey sample*.

### Procedures to examine the prevalence of firesetting in a normative sample

The National Survey dataset was used to examine the prevalence of firesetting in a large normative sample. Initial frequency analyses were run to identify the self-reported incidence of firesetting in a large, normative sample. After this frequency data was examined, the firesetting item was again recoded to 0 (no firesetting) and 1 (firesetting). Cross-Tables were run to investigate prevalence of firesetting comparing males and females in this sample. A logistic regression analysis was conducted to determine if gender was predictive of firesetting in this sample.

The reported rates of firesetting by each race was also investigated using cross-tabs and logistic regression. The purpose of these analyses was to determine if race is associated with firesetting in a randomly selected sample that includes adolescents from all across the United States and several other countries. Most studies on firesetting include individuals from only one geographic area so this data represented a better opportunity to examine the relationship between these two variables.

### Results of analyses examining the prevalence of firesetting in a normative sample

When investigating firesetting utilizing the National Survey dataset (*N* = 1161), it was found that a small percentage of the adolescents reported firesetting behavior (see Table 16) An even lower number of parents/guardians reported their child set fires (*n* = 15). The youth’s report of firesetting was recoded to 0 (no firesetting) and 1 (firesetting) (*n* = 53).

**Table 16:** **Frequency of self-reported firesetting (original coding)**.

Response to “I set fires”	Frequency	Percent	Valid percent	Cumulative percent
Not true	1105	95.2	95.4	95.4
Somewhat or sometimes true	47	4.0	4.1	99.5
Very true or often true	6	0.5	0.5	100.0
Total	1158	99.7	100.0	
Missing	3	0.3		
Total	1161	100.0		

*National survey sample*.

The main focus of this question was to address whether the same difference in the prevalence of firesetting between boys and girls also existed in larger more representative samples. Cross-tabs were run to examine the frequency of firesetting among boys and girls. Boys (*n* = 568) reported 41 firesetting cases (77.36% of the firesetters) while girls (*n* = 537) only had 12 individuals who reported setting fires (see Table 17). A logistic regression indicated that the predicted odds of a juvenile setting a fire decreased by 69% if the individual is female (odds ratio of 0.31, *p* < 0.001; χ^2^ = 14.53; *p* < 0.001; Nagelkerke *R*^2^ = 0.04).

**Table 17:** **Frequency of firesetting by gender in the national survey sample**.

Firesetting reported	Males	Females
Yes (1)	41	12
No (0)	568	537

An initial cross-tabs analysis was run to determine the number of individuals of each race who reported firesetting. A logistic regression was then run to examine the influence of race on firesetting in this sample. The race variable was again recoded to 1 – Caucasian (*n* = 718), 2 – African American (*n* = 227), and 3 – Other (*n* = 216) from the original six groups. See Table 18 for frequency of firesetting by race. The logistic regression indicated no significant difference in the odds of firesetting between races.

**Table 18:** **Frequency of firesetting by race in the national survey sample**.

Firesetting reported	Caucasian	African American	Other race
Yes (1)	35	5	13
No (0)	680	222	203

### Procedure to examine the characteristics of firesetters in a normative sample

The final set of analyses were run to investigate whether known correlates of firesetting would also be related to that behavior in a large normative sample, versus data derived from clinical, inpatient, and outpatient settings.

Initial descriptive information about the parent and child ratings of internalizing, externalizing, and total problems was analyzed to determine which scale scores to include. The academic performance scale (Academic Performance) was also created in the National Survey dataset.

Independent samples *t*-tests were run to examine the group differences between firesetters and non-firesetters on ratings of Internalizing (Internal_raw), Externalizing (External_raw), and Total Problems (TotalProb_raw). Finally, the differences between firesetters and non-firesetters were examined using *t*-tests for academic performance (Academic Performance), attention (Attention_raw), and ADHD symptoms (ADHD_raw). Both adolescent and parent reports were used in the *t*-test analysis examining attention problems and ADHD symptoms.

### Results of analyses examining the characteristics of firesetters in a normative sample

The final research question addressed another gap in the firesetting research which is the lack of studies using large, normative populations rather than clinical samples. Since much of what is known about the characteristics of juvenile firesetters comes from clinical samples, the goal was to determine if some of these characteristics are also associated with firesetting in a more representative sample.

Descriptives were run to determine which of the available variables measuring Internalizing, Externalizing, and Total Problems should be used for the regressions. Similar to the firesetting variable, adolescent’s self-reported more internalizing problems than their parents (see Table 19). The decision was made to utilize the adolescent’s self-reported ratings of internalizing, externalizing, and total problems for multiple reasons. Primarily, the items on these scales measure behaviors or thoughts that a parent or guardian may not be aware their child is having. Additionally, the difference in frequency on the firesetting item illustrates that parents may be under-reporting these types of issues and the adolescent’s rating may be a more accurate measure of the child’s functioning.

**Table 19:** **Comparison of self-report and parent ratings for problem variables**.

	Internal YSR	Internal parent	External YSR	External parent	Total YSR	Total parent
*M*	10.48	6.74	10.38	7.77	37.64	25.63
SD	7.90	6.35	7.49	8.12	22.32	21.17
*N*	1159	1160	1159	1160	1160	1160

Independent *t*-tests were conducted to determine if there were significant differences between firesetters and non-firesetters reports of Internalizing, Externalizing, and Total Problems. Significant differences were found in all three areas between the firesetter and non-firesetter groups. The firesetter group reported more Internalizing, Externalizing, and Total Problems than the adolescents who reported no firesetting. The results were statistically significant. See Table 20 for results.

**Table 20:** **Internalizing, externalizing, and total problem means by firesetting group**.

	Firesetting		*t*	*df*
	Yes	No	
Internalizing problems	16.66 (10.94)	10.19 (7.61)	−4.26**	54.45
Externalizing problems	18.32 (10.56)	10.01 (7.10)	−5.67**	54.28
Total problems	60.47 (32.67)	36.59 (21.11)	−5.27**	54.10

*Standard deviations appear in parentheses below means*.

National survey sample.*p ≤ 0.05; **p ≤ 0.01; ***p ≤ 0.001.

Lastly, analyses were conducted to determine if differences exist between firesetters and non-firesetters on reports of academic performance, attention, and ADHD symptoms. Independent sample *t*-tests were conducted comparing firesetters to non-firesetters in the areas of academic performance (Academic Performance), parent and child reported attention (Attention_CBCL and Attention_YSR), and symptoms of ADHD reported by the parent and child (ADHD_CBCL and ADHD_YSR). Statistically significant differences between firesetters and non-firesetters were evident in reported academic performance as measured by grades and both the parent and child’s reports of attention problems and ADHD symptoms (see Table 21).

**Table 21:** **Attention, ADHD, and academic performance means by firesetting group**.

	Firesetting		*t*	*df*
	Yes	No	
Academic performance	3.09 (0.66)	3.26 (0.48)	2.50*	1048
Attention problems-youth	7.68 (3.58)	5.01 (3.24)	−5.84***	1154
Attention problems-parent	6.43 (4.63)	3.78 (3.67	−4.11***	55.17
ADHD symptoms-youth	6.34 (3.17)	4.51 (2.82)	−4.58***	1154
ADHD symptoms-parent	4.66 (3.62)	3.12 (3.00)	−3.05**	55.38

*Standard deviations appear in parentheses below means*.

*p ≤ 0.05; **p ≤ 0.01; ***p ≤ 0.001.

## Discussion

The purpose of this study was to identify school related predictors of juvenile firesetting and examine the prevalence of firesetting in a large dataset consisting of children from non-clinical settings.

### Academic performance and firesetting

Results indicate that gender and academic performance are significant predictor variables. Although being male increases the likelihood that a child will set a fire by 70% when compared with a female child of the same age and race, it was also found that children and adolescents who report poorer academic performance are more likely to set fires when controlling for gender and race. Children with failing performance in the four main academic areas are much more likely to set fires than their peers who are academically successful. The results of independent samples *t*-test using the National Survey sample also found a significant difference in academic performance between firesetters and non-firesetters.

A child’s attitude toward school is more predictive of firesetting than academic performance. Interestingly, when attitude toward school is entered as a control variable, academic performance is no longer a significant predictor. This final model explained 13.8% of the variance as compared to the initial model using only academic performance (8.6%). These findings generated a new prediction model for predicting firesetting with school related performance and attitude. Children and adolescents’ perception of their academic performance, truancy, and disobedience at school, along with their performance as measured by grades is predictive of firesetting. Although the assumption cannot be made that a child with both low academic performance and a poor attitude toward school will set fires, it certainly encourages teachers and parents to pay attention to an adolescent who is displaying behavior problems at school, skipping school, and has poor grades.

### Attention problems, ADHD, and firesetting

Results of this study indicate that attention problems and ADHD symptoms as reported by the child or parent were predictive of firesetting. Four regressions were run using the parent’s ADHD and Attention scale raw score as well as the adolescent’s ADHD and Attention scale score. The models explained 8.2–9.2% of the variance, respectively, with the youth’s self-report serving as a better predictor of firesetting. Children who displayed higher levels of inattention, hyperactivity, and symptoms of ADHD were more likely to set fires. This was found to be true for both the children in the Factor Analysis and National Survey data sets. These findings confirm previous research from clinical settings suggesting hyperactivity and impulsivity play a role in firesetting. Our study found a correlation between firesetting and ADHD, but a lower percentage than the fire setter intervention program in San Diego County, California, where between 20 and 40% of the children had been diagnosed with ADD (26) and another study (12) that found only 20%. Further research is needed to explore the correlation of firesetting and ADD/ADHD and it would be helpful to determine, if management of ADD/ADHD symptoms can minimize firesetting as well.

### Prevalence and characteristics of firesetters in a normative sample

A smaller percentage (4.5%) of children in the normative sample reported firesetting than in the sample containing children with elevated levels of problems. Significantly more males than females report setting fires. Gender is highly predictive of firesetting with males being 69% more likely to set fires than their female peers. This is consistent with other researcher findings in a variety of settings; so, it appears that regardless of the population, gender plays a significant role in a child’s behavior involving fire. Internalizing problems, externalizing problems, and total problems were all associated with firesetting even in a normative sample. Firesetters reported more problems in all these areas as well as academic problems, attention problems, and symptoms of ADHD. In consideration of the findings with the National Survey dataset and their consistency with findings from clinical settings, it is possible that many children who set fires also display enough other problematic behaviors that they end up being referred to a mental health or other professional. This would explain why the results of this study are consistent with those examining samples from clinical settings.

### Limitations of the study

This study had several limitations that should be considered. The composition of the Factor Analysis data set, although more representative of the general population than many firesetting studies, still consists of adolescents with higher levels of reported problems than an “average” child. Due to this limitation, care should be taken when generalizing the findings from the Factor Analysis sample to other populations. An additional limitation is the small number of firesetters in the National Survey sample. The usage of one item as the measure of firesetting could also be considered a limitation. A child’s interpretation of “I set fires” may not include match play or fire play when items or objects were not burned. Use of the dichotomous dependent variable (firesetter or non-firesetter) also restricted the type of data analysis that could be performed.

## Conclusion

The purpose of this study was to identify academic and attention characteristics of juvenile firesetters and determine if these were predictive of firesetting in order to address gaps in the existing research. Additionally, due to the availability of a large normative dataset, the study was also designed to examine the prevalence of firesetting and whether characteristics known to be associated with firesetting in clinical samples are also related when looking at a more generalized population of children.

The findings of this study serve to support and enhance existing knowledge about juvenile firesetting. It is concluded that academic problems and poor school attitude were predictive of firesetting and increased the odds of child or adolescent setting fires. Analysis of the relationship between gender and firesetting confirmed that boys were much more likely to set fires than their female peers. Analysis of the National Survey sample confirmed that firesetting is a behavior predominantly displayed by boys and associated with internalizing and externalizing behavior problems. The odds of being a firesetter increased when a child reported more problems in these areas as well as lower academic performance, a poor attitude to school and attention problems.

Firesetting is a very dangerous behavior that results in the loss of lives every year. The focus of this study was placed on academic and attention variables because children spend the majority of time at school where teachers and school psychologists can identify problems related to school functioning that are predictive of firesetting. Additionally, many fire service professionals work with children who have set fires and although they may not have access to mental health records they can coordinate with parents and teachers to gather information about academic and attention risk factors to help better assess the adolescent’s risk level and design intervention. It is our hope that the findings of this study will assist practitioners in the schools and fire service in providing better services and also encourage other researchers to study the problem as well.

## Conflict of Interest Statement

The authors declare that the research was conducted in the absence of any commercial or financial relationships that could be construed as a potential conflict of interest. The Associate Editor Ariel Tenenbaum declares that, despite being affiliated to the same institution as author Joav Merrick, the review process was handled objectively and no conflict of interest exists.
